# Phosphodiesterase 8a Supports HIV-1 Replication in Macrophages at the Level of Reverse Transcription

**DOI:** 10.1371/journal.pone.0109673

**Published:** 2014-10-08

**Authors:** Thijs Booiman, Viviana Cobos Jiménez, Karel A. van Dort, Angélique B. van 't Wout, Neeltje A. Kootstra

**Affiliations:** Department of Experimental Immunology, Sanquin Research, Landsteiner Laboratory and Center for Infection and Immunity (CINIMA) at the Academic Medical Center of the University of Amsterdam, Amsterdam, The Netherlands; Florida Atlantic University, United States of America

## Abstract

**Background:**

HIV-1 infected macrophages play a key role in HIV-1 infection. Even during anti-retroviral treatment, macrophages keep producing virus due to suboptimal tissue penetration and reduced efficacy of antiretrovirals. It is therefore of major importance to understand which host factors are involved in HIV-1 replication in macrophages. Previously, we have shown that genetic polymorphisms in phosphodiesterase 8a (PDE8A) are strongly associated with HIV-1 replication in these cells. Here we analyzed the mechanism and regulation of PDE8A in HIV-1 replication in macrophages.

**Results:**

PDE8A mRNA expression strongly increases upon differentiation of monocytes into macrophages, which corresponds to the increased susceptibility of mature macrophages to HIV-1. In parallel, expression of microRNA miR-145-5p, predicted to target PDE8A mRNA, strongly decreased. The interaction of miR-145-5p with the 3′ UTR of PDE8A mRNA could be experimentally validated, suggesting that indeed miR-145-5p can regulate PDE8A expression levels. Knockdown of PDE8A in macrophages resulted in a decrease in total HIV-1 replication and proviral DNA levels. These observations confirm that PDE8A regulates HIV-1 replication in macrophages and that this effect is mediated through early steps in the viral replication cycle.

**Conclusions:**

PDE8A is highly expressed in macrophages, and its expression is regulated by miR-145-5p. Our findings strongly suggest that PDE8A supports HIV-1 replication in macrophages and that this effect is mediated at the level of reverse transcription.

## Introduction

Macrophages play a key role in HIV-1 infection. They are among the first cells that encounter HIV-1 upon transmission and once infected they facilitate spread of the virus to CD4+ T-cells [1]–[7]. Infected macrophages are relatively resistant to HIV-1 induced apoptosis and can efficiently evade host immunity [8], [9]. Due to the ability of these cells to migrate into the tissue, the virus is disseminated throughout the body [10]–[14]. The introduction of combined antiretroviral therapy (cART) has reversed the fatal outcome of HIV-1 infection. Nonetheless, infected macrophages remain a source of residual virus production during treatment due to the low efficacy of cART, combined with the suboptimal tissue penetration of the drugs [10]–[15]. In addition to their contribution to the viral reservoir, HIV-1 infected macrophages play a crucial role in several AIDS related pathologies such as HIV-1 associated dementia, AIDS-related non-Hodgkin lymphoma and cardiovascular diseases [16]–[19]. It is therefore of major importance to specifically target HIV-1 replication in macrophages. *In vitro*, freshly isolated monocytes are almost refractory to HIV-1 infection and become permissive during differentiation into macrophages [20]–[23]. The differential susceptibility of monocytes and macrophages to HIV-1 is at least in part regulated by host factors [24]–[29]. Previously, we identified several host-genetic factors that were strongly associated with *in vitro* HIV-1 replication in macrophages [30], [31]. We observed that four SNPs in the phosphodiesterase 8A (*PDE8A*) gene were highly associated with HIV-1 replication [31]. This suggests that PDE8A plays an important role in HIV-1 replication in macrophages. Indeed, a recent RNAi screen identified PDE8A as a HIV-1 dependency factor [32]. PDE8A is a phosphodiesterase that hydrolyzes cyclic adenosine monophosphate (cAMP) to AMP [33]. Several studies indeed have shown a role for cAMP in HIV-1 infection and it was demonstrated that cAMP can affect several steps of the viral replication cycle ranging from entry to proviral transcription [34].

Here we analyzed the regulation of *PDE8A* expression during monocyte differentiation and cytokine polarization. Furthermore, we investigated the effect of PDE8A knockdown on HIV-1 replication.

## Results

### PDE8A expression during differentiation and polarization of macrophages

**Figure 1:**
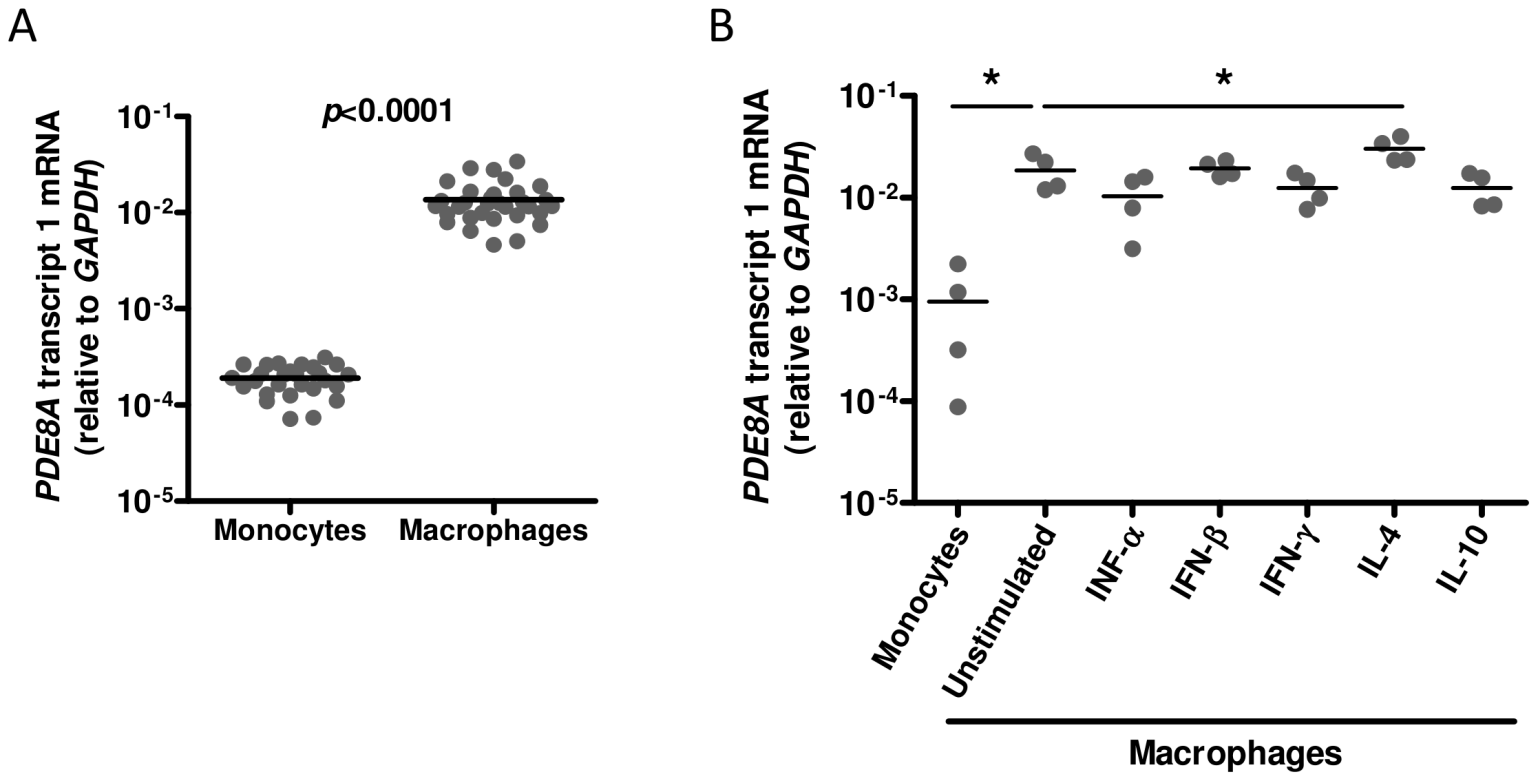
PDE8A expression during macrophage differentiation and polarisation. (A) *PDE8A* mRNA expression in freshly isolated monocytes and macrophages cultured for 7 days from 32 donors. (B) *PDE8A* mRNA expression in freshly isolated monocytes and macrophages from 4 donors cultured in the absence or presence of IFN-α (250 U/ml), IFN-β (250 U/ml), IFN-γ (2501U/ml), IL-4 (10 ng/ml), or IL-10 (100 U/ml) for 7 days. Statistical differences were analyzed using a paired T test: *p<0.05, **p<0.01, ***p<0.001.

Previously, we showed that genetic polymorphisms in the *PDE8A* gene are strongly associated with HIV-1 replication in macrophages [30], [31]. *In vitro*, monocytes become highly susceptible to HIV-1 after differentiation into macrophages [20]–[23]. To determine whether PDE8A plays a role in the differential susceptibility of macrophages during differentiation, *PDE8A* mRNA expression was analyzed in freshly isolated monocytes and macrophages derived from 31 healthy blood donors. We observed on average a 70-fold increase in *PDE8A* expression upon differentiation of monocytes into macrophages, which suggests that PDE8A may be one of the factors that support the increased HIV-1 susceptibility of macrophages during differentiation (Figure 1A).

HIV-1 replication is strongly inhibited in macrophages stimulated with IFN-α, IFN-β, IFN-γ, IL-4, and IL-10 [22], [24], [35]–[41]. We analyzed whether differential expression of PDE8A during cytokine activation of macrophages may contribute to the decreased susceptibility of these cells to HIV-1 infection. Again, *PDE8A* mRNA expression increased during differentiation of monocytes into 5-day old macrophages (Figure 1B). Differentiation of monocytes in the presence of IFN-α, IFN-β, IFN-γ, or IL-10 did not change *PDE8A* expression, whereas the addition of IL-4 increased *PDE8A* expression (Figure 1B). This indicates that the restriction of HIV-1 replication observed in cytokine stimulated macrophages is not mediated by differential regulation of PDE8A expression.

### PDE8A expression is regulated by miR-145-5p during differentiation of macrophages

**Figure 2:**
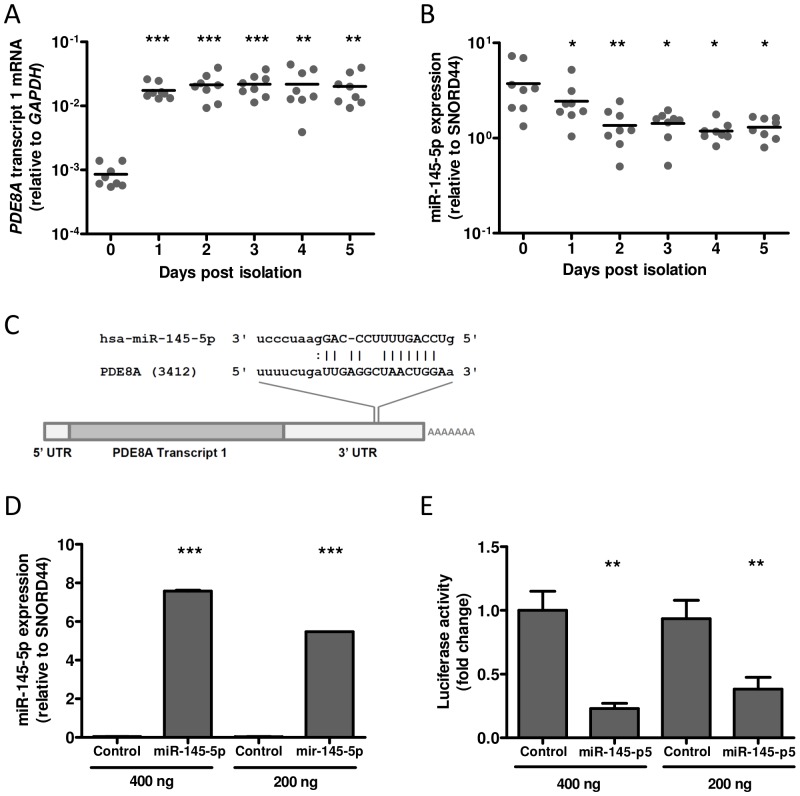
Regulation of PDE8A expression by miR-145-5p during differentiation of monocytes into macrophages. Confirmation of *PDE8A* mRNA (A) and miR-145-5p (B) expression during differentiation of monocytes into macrophages obtained from 8 donors. *PDE8A* mRNA or miR-145-5p expression during differentiation was compared with the expression at day 0 by using a paired T test: *p<0.05, **p<0.01, ***p<0.001. (C) Schematic overview of the *PDE8A* mRNA transcript 1 including the miR-145-5p binding site between base pair 3412 and 3435. (D) Expression levels of miR-145-5p determined by qPCR in HEK293T cells 2 days after transfection with an empty vector control or a vector containing a miR-145-5p expression cassette (n = 2, mean and SD). (E) Direct interaction between miR-145-5p and its target sequence in the *PDE8A* mRNA transcript was tested by co-transfection of p-hEF1a-miR-145-5p or an empty vector control and p-hEF1a-Luc-3′UTR PDE8A. Luciferase activity was measured 2 days post-transfection (n = 3, mean and SD). Statistical differences were analyzed using an un-paired T test: *p<0.05, **p<0.01, ***p<0.001.

miRNAs are known to play a key role in post-transcriptional regulation of gene expression and recently it was observed that miRNAs are differentially expressed during maturation of monocytes and polarization of macrophages by cytokines [42], [43]. Therefore, we studied whether PDE8A expression is regulated by miRNAs during macrophage differentiation. miRNAs that target *PDE8A* mRNA were selected from miRNA-mRNA interactions that have been predicted and reported in the Ingenuity Knowledge Base, TarBase and miRecords and TargetScan [44]–[46]. Potential miRNAs regulating PDE8A expression were identified by pairing gene and miRNA expression data from monocytes and macrophages cultured for 5 days [43], [47]. Of the 190 miRNAs that potentially could target *PDE8A*, we found that miR-145-5p, miR-577 and miR-1280 were significantly downregulated in 5-day cultured macrophages as compared to monocytes, while *PDE8A* expression was upregulated (Table 1). To confirm these results, we determined *PDE8A* and miRNA expression during differentiation of monocytes into macrophages in 8 additional donors. *PDE8A* expression increased significantly during the first day of differentiation and this effect was maintained during differentiation (Figure 2A). In contrast, expression of miR-145-5p decreased significantly during the first day of differentiation and miR-145-5p remained low during subsequent differentiation (Figure 2B). Expression of miR-577 could not be confirmed by qPCR, whereas down-regulation of expression of miR-1280 was not observed (data not shown). miR-145-5p is predicted to target the 3′UTR of the *PDE8A* mRNA transcript (Figure 2C). To confirm whether miR-145-5p can regulate PDE8A expression, we constructed a miR-145-5p overexpression vector and a luciferase reporter vector in which the 3′UTR of the *PDE8A* mRNA transcript was cloned into the 3′UTR of the luciferase gene. First, HEK293T cells were transfected with different concentrations of the miR-145-5p vector and miR-145-5p expression levels were analyzed 2 days after transfection by qPCR. We observed a dose dependent increase of miR-145-5p expression as compared to the empty vector control (Figure 2D). Next, we performed co-transfections of the miR-145-5p overexpression vector and the luciferase reporter vector containing the predicted miR-145-5p target sequence. We observed that miR-145-5p is able to down regulate luciferase activity in a dose dependent manner (Figure 2E). This suggests that miR-145-5p is indeed able to target the 3′UTR of *PDE8A* mRNA thereby regulating protein expression levels.

**Table 1:** Potential miRNAs targeting PDE8A.

miRNA ID	Fold Change miRNA Day 5 vs Day 0 #	Source	Confidence	Gene Symbol	Fold Change mRNA Day 5 vs Day 0 #
**hsa-miR-145-5p**	0.102	TargetScan Human	High (predicted)	PDE8A	2.794
**hsa-miR-1280**	0.098	TargetScan Human	High (predicted)	PDE8A	2.794
**hsa-miR-577**	0.128	TargetScan Human	High (predicted)	PDE8A	2.794

# Fold change in expression in which day 5 is compared to day 0; >1 is an increase and <1 is a decrease in expression.

### PDE8A affects HIV-1 replication at the level of reverse transcription

**Figure 3:**
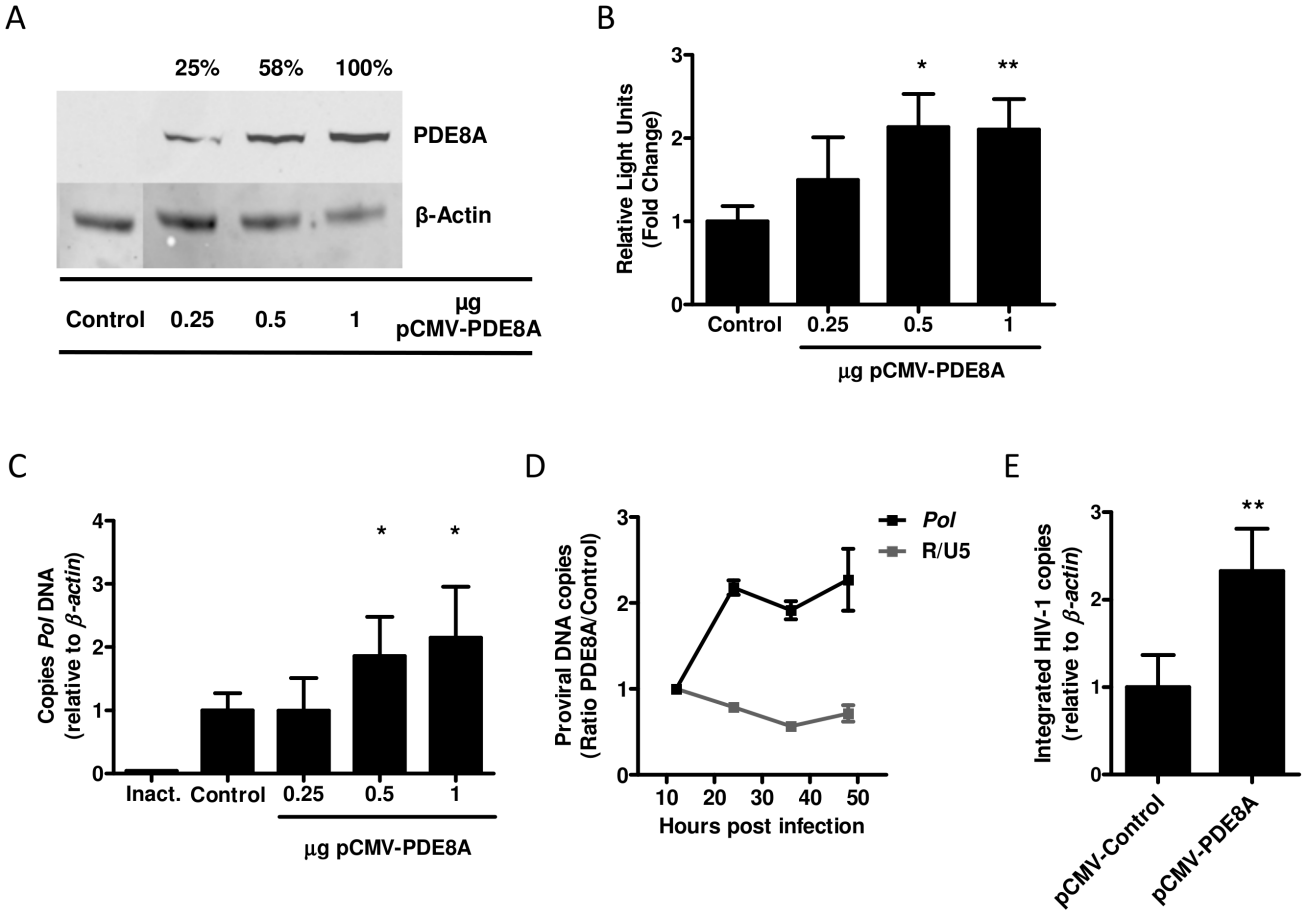
PDE8A supports HIV-1 replication at the level of reverse transcription. (A) HEK293T cells were transfected with different concentrations of pCMV6-PDE8A or an empty vector control. PDE8A expression was analyzed forty-eight hours after transfection by western blot. (B) HEK293T cells were transfected with different concentrations of pCMV6-PDE8A or an empty vector control and infected with VSV-G/NL4-3.Luc 48 hours after transfection. Luciferase activity was measured 2 days after infection (n = 3, mean and SD). (C) HEK293T cells were transfected with different concentrations of pCMV6-PDE8A or an empty vector control and infected with VSV-G/NL4-3-Ba-L 48 hours after transfection. Proviral DNA levels were analyzed forty-eight hours after infection by qPCR. Proviral DNA levels are expressed relative to the control after correction for β-actin levels in the same sample (n = 3, mean and SD). (D) HEK293T cells were transfected with 1 µg of pCMV6-PDE8A or 1 µg of empty vector control and infected with VSV-G/NL4-3-Ba-L 48 hours after transfection. *R/U5* and *Pol* proviral DNA levels were analyzed by qPCR during 48 hours post-infection. *R/U5* and *Pol* levels are expressed as the ratio of the expression in cells transfected with pCMV-PDE8A divided by the expression in cells that were transfected with the empty vector control and corrected for β-actin levels (n = 2, mean and SD). (E) HEK293T cells were transfected with 1 µg of pCMV6-PDE8A or 1 µg of the empty vector control and infected with VSV-G/NL4-3-Ba-L 48 hours after transfection. Integrated HIV-1 copies were determined 48 hours post infection by Alu-PCR and expressed relative to the control after correction for β-actin levels in the same sample (n = 2, mean and SD). Statistical differences were analyzed using an un-paired T test: *p<0.05, **p<0.01, ***p<0.001.

To determine which step of the replication cycle is affected by PDE8A we overexpressed a myc-tagged PDE8A in HEK293T cells and infected these cells with a VSV-G/NL4-3.Luc reporter virus. Overexpression of PDE8A resulted in a dose-dependent increase in luciferase activity (Figure 3A and 3B). This finding confirms our previous results and indicates that PDE8A supports HIV-1 replication. Next, we analyzed the effect of PDE8A overexpression on early steps of the viral replication cycle by analysis of proviral DNA levels during 48-hours post infection. HEK293T cells overexpressing PDE8A showed a dose dependent increase in *Pol* proviral DNA at 48-hours post infection (Figure 3C). The effect of PDE8A on *Pol* proviral DNA levels was already mediated within the first 24-hours post infection, whereas levels of strong stop proviral DNA as determined by *R/U5* qPCR were not affected (Figure 3D). Furthermore, PDE8A overexpression resulted in increased levels of integrated HIV-1 provirus as determined by Alu-PCR (Figure 3E). This indicates that PDE8A supports HIV-1 replication at the level of reverse transcription.

### PDE8A supports HIV-1 replication in monocyte-derived macrophages

**Figure 4:**
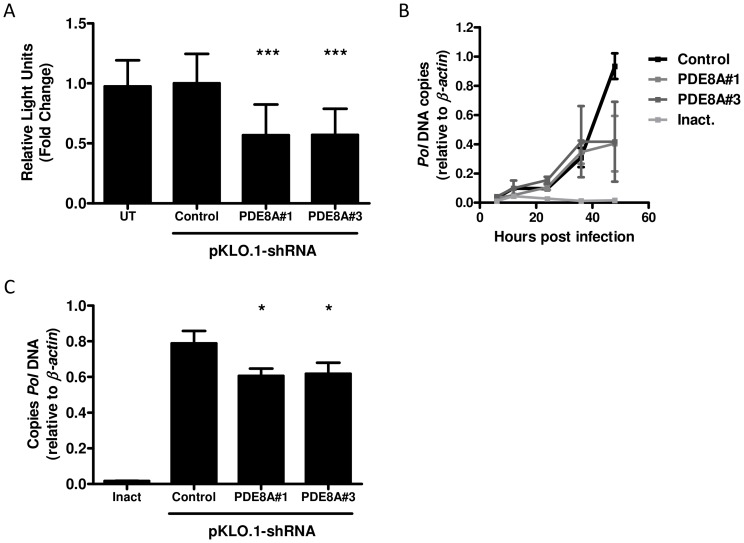
The effect of PDE8A knockdown on HIV-1 replication in macrophages. (A) To analyze the effect of PDE8A knockdown on HIV-1 replication in macrophages, monocytes from 4 donors were cultured for 5 days and transduced with lentiviral vectors expressing shRNA control or two shRNA's targeting PDE8A. Forty-eight hours after the lentiviral transduction, macrophages were infected with VSV-G/NL4-3.Luc and luciferase activity was measured at day 2 after infection. The results are given as the mean fold luciferase expression from 4 donors, relative to the medium control. UT: Untransduced control. Statistical differences were analyzed by mixed linear model, repeated covariance: diagonal (IBM SPSS Statistics 20). (B) To determine the effect of PDE8A knockdown on *Pol* proviral DNA levels, monocytes in which PDE8A was downregulated by lentiviral transduction were infected with VSV-G/NL4-3-Ba-L. *Pol* proviral DNA levels were analyzed by qPCR during 48 hours post-infection and expressed relative to β-actin. Results are given as mean and sd from 2 donors. (C) Monocytes in which PDE8A was downregulated by lentiviral transduction were infected with VSV-G/NL4-3-Ba-L. Two days post-infection, proviral DNA levels were analyzed by qPCR for *Pol*. Proviral DNA levels are expressed relative to the control after correction to β-actin for input. Results shown are representative for 3 donors. Inact.: Inactivated virus. Statistical differences were analyzed using a paired T test: *p<0.05, **p<0.01, ***p<0.001.

To determine the role of PDE8A in HIV-1 replication in macrophages, we analyzed the effect of PDE8A knockdown by shRNAs on the HIV-1 replication cycle in these cells. We selected two lentiviral vectors that were able to knockdown PDE8A expression (Figure S1A). Macrophages were transduced with the lentiviral vectors expressing either the shRNAs targeting PDE8A or a control shRNA which reduced *PDE8A* mRNA in most donors (Figure S1B). Subsequent infection with a VSV-G/NL-4-3.Luc reporter virus, resulted in a significant decrease in luciferase activity in the PDE8A knockdown cells (Figure 4A), demonstrating that HIV-1 replication in macrophages is supported by PDE8A. Next, the effect of PDE8A on proviral DNA synthesis was analyzed in macrophages. Knockdown of PDE8A expression resulted in a decrease in *Pol* proviral DNA levels at 48-hours post-infection (Figure 4B and 4C). This further confirms that PDE8A supports HIV-1 replication in macrophages and that this effect is mediated at the level of reverse transcription.

## Discussion

The HIV-1 genome encodes only 15 proteins and therefore HIV-1 is heavily dependent on host proteins for its replication. Host cell proteins that are required for efficient replication of HIV-1 in macrophages are attractive targets for new therapeutic approaches and therefore it is crucial to better understand the interaction between HIV-1 and these host factors. In our previous work, we have identified polymorphisms in PDE8A that are strongly associated with PDE8A expression and *in vitro* HIV-1 replication in macrophages [30], [31]. Here, we showed that *PDE8A* expression is highly upregulated during differentiation of monocytes into macrophages and that miR-145-5p may be involved in the regulation of PDE8A expression during differentiation. We demonstrated that PDE8A supports HIV-1 replication in macrophages and that this effect is mediated at the level of reverse transcription. These data suggest that PDE8A plays an important role in HIV-1 susceptibility of macrophages during differentiation [20]–[23].

PDE8A is a phosphodiesterase that regulates cAMP levels by hydrolyzing cAMP to AMP [33]. In the context of HIV-1 infection cAMP has been described as having opposing effects on the different steps of the viral replication cycle, which seems to be partially cell type dependent (reviewed by [34]). Here we show that increasing PDE8A expression levels are associated with higher concentrations of HIV-1 proviral DNA and subsequent higher levels of integrated HIV-1 provirus. This suggests that PDE8A supports HIV-1 replication at the level of reverse transcription. The increased proviral DNA synthesis in macrophages may be the result of low cAMP levels mediated by high PDE8A expression levels. Indeed, Thivierge *et al*. and Hayes *et al.* have shown that high cAMP levels decrease viral replication in macrophages [48], [49]. Thivierge *et al.* attributed this effect to decreased expression of co-receptor CCR5 in cells with high cAMP levels [48], however they did not determine the effect of cAMP on the different steps in viral replication and only analyzed virus production in the culture supernatant. Here we used a VSV-G pseudotyped reporter virus to circumvent CD4 and CCR5 mediated entry and demonstrated that PDE8A affects HIV-1 replication independent of CCR5 expression levels. Hayes *et al.* demonstrated that high cAMP levels decreased HIV-1 mRNA expression and viral replication in macrophages, but this may have been the result of decreased proviral DNA synthesis as the effect of high cAMP levels on proviral DNA synthesis was not determined in this study [49].

PDE8 isoforms exhibit an extremely high affinity for cAMP and it is therefore suggested that PDE8 enzymes have an important role in protecting any associated protein from fluctuations in basal cAMP concentrations [50]. Besides the direct effect of PDE8A on total cellular cAMP levels, it is also possible that PDE8A affects the replication cycle via a binding partner like RAF-1. It has been demonstrated that RAF-1 plays a role in HIV-1 infection in dendritic cells, and that it is required for the induction of proviral transcription [51]. PDE8A associates with RAF-1 and prevents its inactivation by phosphorylation by protein kinase A (PKA) [52]. However, we observed that knockdown of RAF-1 did not affect the ability of PDE8A to support HIV-1 replication (data not shown). This indicates that the effect of PDE8A on HIV-1 replication described here is not mediated through RAF-1.

## Conclusions

Here we demonstrated that PDE8A supports HIV-1 replication in macrophages at the level of reverse transcription. PDE8A is highly expressed in macrophages and can be regulated by miR-145-5p during differentiation. Macrophages are considered to be a source of residual viral replication even under cART. Since PDE enzymes are considered promising targets for pharmacological intervention [53]–[56], blocking of PDE8A may specifically interfere with HIV-1 replication in primary macrophages.

## Methods

### Ethics statement

This study has been conducted in accordance with the ethical principles set out in the declaration of Helsinki, and was approved by the Medical Ethics Committee of the Academic Medical Center and the Ethics Advisory Body of the Sanquin Blood Supply Foundation in Amsterdam, The Netherlands. Written informed consent was obtained from all participants.

### Cell culture

Peripheral blood mononuclear cells (PBMC) were obtained from buffy coats from healthy blood donors. PBMC were isolated by Ficoll-Isopaque density gradient centrifugation and subsequently monocytes were isolated by magnetic-activated bead technology using CD14+ magnetic microbeads (Miltenyi Biotec, Bergisch Gladbach, Germany). Monocytes were differentiated in Iscove's modified Dulbecco medium (IMDM) supplemented with 10% (v/v) heat-inactivated human pool serum (HPS), penicillin (100 U/ml), streptomycin (100 µg/ml) and cyproxin (5 µg/ml) in the presents or absence of IFN-α (250 U/ml), IFN-β (250 U/ml), IFN-γ (250 U/ml), IL-4 (10 ng/ml), or IL-10 (100 U/ml) and maintained in a humidified 10% CO_2_ incubator at 37°C. HEK293T cells were cultured in Dulbecco's Modified Eagle Medium without Hepes (DMEM) (Lonza, Basel, Switzerland) supplemented with 10% (v/v) inactivated fetal calf serum (FCS), penicillin (100 U/ml) and streptomycin (100 µg/ml), and maintained in a humidified 10% CO_2_ incubator at 37°C [57].

### Plasmids

For PDE8A overexpression and testing knockdown efficiency of the shRNAs targeting PDE8A, a vector expressing a MYC-tagged PDE8A under the control of the CMV promotor (Origene, Rockville, MD, USA) was used. The vector containing the miR-145-5p overexpression cassette was created by cloning the stem-loop sequence of human miR-145-5p (miRBase accession number MI0000461) including approximately 100 base pairs up- and down-stream of the stem-loop in a HIV-1 based lentiviral expression vector pLV-hEF1a in which expression is driven by the human Elongation Factor 1 alpha promotor (p-LV-hEF1a-miR-145-5p) [58], [59]. The luciferase reporter vector containing the 3′UTR of the PDE8A mRNA transcript, which is targeted by miR-145-5p was constructed as follows: The miR-145-5p target sequence from 3′UTR of the PDE8A mRNA transcript, including flanking regions of 40 base pairs, was cloned into the 3′UTR sequence of a luciferase reporter gene in a pLV-pHEF1a-Luciferase vector in which expression is driven by the human Elongation Factor 1 alpha promotor (p-hEF1a-Luc-3′UTR PDE8A) [58], [59]. Primers used for cloning are listed in Table S1.

### Virus production

YU2 and VSV-G pseudotyped NL4-3.Luc.R-E- luciferase reporter virus were produced by transfection of respectively pYU2 or pNL4-3.Luc.R-E- with pCMV-VSV-G in HEK293T cells. Lentiviral vectors were produced by co-transfection of pLV-CMV-GFP [58], [59] or pLKO.1 constructs expressing shRNA candidates from the MISSION TRC-Hs 1.0 library (PDE8A TRCN48874, PDE8A TRCN48876 and control SHC001; Sigma-Aldrich St. Louis, MO, USA) [60] and pCMV-VSV-G, pMDLgp and pRSV-Rev in HEK293T cells [61]. Transfections were performed with the calcium phosphate method. In short, plasmid DNA was diluted in 0.042M HEPES containing 0.15M CaCl_2_, subsequently mixed with an equal volume of 2× HEPES buffered saline pH 7.2, incubated at room temperature for 15 min and added to the culture medium. After 24 h incubation in a humidified 3% CO_2_ incubator at 37°C, the culture medium was replaced and cultures were continued at 10% CO_2_ at 37°C. Virus was harvested at 48 and 72 h after transfection and passed through a 0.22 µm filter. HIV-1 virus titers were quantified by determining the TCID50 on TZMBL, HEK293T or U87 cells [62]. Lentiviral vector supernatant was concentrated by centrifugation at 50,000xg for 2 hours directly after harvesting. Equal amounts of lentiviral vector as determined by p24 ELISA were used to inoculate target cells.

### Infection of 293T cells

Cells were transfected as described previously with pCMV6-PDE8A [31]. Twenty-four hours post transfection cells were detached using trypsin (Invitrogen, Carlsbad, CA) and seeded in 24-wells plates to determine proviral DNA levels and in 96-wells plates to determine total HIV-1 replication. To determine proviral DNA levels, cells were inoculated at a multiplicity of infection (MOI) of 0.01 with VSV-G pseudotyped NL4-3-B-aL which was treated with DNAse (200 ng/ml) (RQ1; Promega Corp., Madison WI, USA) for 45 min at 37°C in medium containing 6 mM MgCl_2_. As a control, a heat-inactivated (30 min; 56 C) inoculum was used. During 48-hours hours post infection, total DNA was isolated using the L6 method [63]. Proviral DNA was measured by qPCR using primers and probes detecting *R/U5* or *Pol* products (Table S1). To determine the effect of PDE8A on total HIV-1 replication, cells were inoculated at an MOI of 0.01 with VSV-G pseudotyped NL4-3.Luc.R-E- luciferase reporter virus. Forty-eight hours post infection, luciferase activity was determined as a measure for HIV-1 replication, by adding 25 µl substrate (0.83 mM ATP, 0.83 mM d-luciferin (Duchefa, Haarlem, The Netherlands), 18.7 mM MgCl_2_, 0.78 µM Na_2_H_2_P_2_O_7_, 38.9 mM Tris pH 7.8, 0.39% (v/v) glycerol, 0.03% (v/v) Triton X-100, and 2.6 µM dithiothreitol) directly to the culture medium. Luminescence was measured for 1 s per well using a luminometer (Berthold, Bad Wildbad, Germany).

### Lentiviral vector transduction and HIV-1 infection of primary cells

Monocytes were cultured for 5 days and transduced with lentiviral vectors expressing shRNA control or two shRNA's targeting PDE8A. Medium was replaced with IMDM without serum or antibiotics and two hours later, macrophages were inoculated with lentiviral vectors at a MOI of 10. Sixteen hours post transduction, medium was replaced with IMDM supplemented with 10% (v/v) heat-inactivated human pool serum (HPS), penicillin (100 U/ml), streptomycin (100 µg/ml) and cyproxin (5 µg/ml). Forty-eight hours post transduction, macrophages were infected with VSV-G pseudotyped luciferase reporter virus at a MOI of 0.2 to analyze total replication. In addition, macrophages were inoculated at a MOI of 0.012 with DNAse-treated YU2 to analyze the effect on proviral DNA levels. Forty-eight hours post transfection luciferase activity was measured as described above.

### Nucleic acid isolation and quantitative PCR

Total RNA was extracted using the High Pure RNA Isolation kit (Roche, Basel, Switzerland) or TriPure Isolation Reagent (Roche). Reverse transcription of mRNA was performed using the Roche Transcriptor First Strand cDNA Synthesis kit (Roche) using a maximum of 1 µg total RNA and oligo(dT) primers. Reverse transcription of miRNAs was performed using the qScript microRNA cDNA synthesis kit (Quanta BioScineces Inc., Gaithersburg, MD). Resulting cDNA was used for quantitative PCR (qPCR) analysis using SYBR Green I Master or Probes Master (Roche) and target specific primers (Table S1). For quantification of miR-145-5p and SNORD44 control, specifically designed primers (Table S1) were used in combination with the PerfeCTa Universal PCR Primer, including the Human positive control (SNORD44) primers (Quanta BioSciences Inc.). Integrated HIV-1 DNA was measured by two-step Alu-PCR as described previously [51]. In short, during the first step Alu-LTR sequences were amplified with an HIV-1-specific primer (LTR R region) and a primer that anneals to the abundant genomic Alu repeats. The HIV-1-specific primer was extended with a marker region at the 5′ end, which was used for specificity in the second-round PCR. The second step consisted out of a nested quantitative real-time PCR in which PCR products from the first step were amplified with primers annealing to the aforementioned marker region and a HIV-1 specific primer (LTR U5 region) (Table S1). To account for the signal contributed by unintegrated HIV-1 DNA, the first-round PCR was also done with the HIV-1-specific primer alone as a control. HIV-1 integration was normalized relative to β-actin DNA. qPCRs were performed on a Lightcycler 480 II (Roche) and gene expression values were obtained using Roche's LightCycler relative quantification software (release 1.5.0).

### Western blot analysis

The effect of PDE8A overexpression and subsequent knockdown by shRNAs on protein levels was analyzed by western blot. Two days post transfection, HEK293T cells were lysed in RIPA-buffer (150 mM NaCl, 1% Triton X-100, 0.5% sodium deoxycholate, 0.1% SDS, 50 mM Tris, pH 8.0) containing Complete EDTA free protease inhibitor (Roche). After adding NuPAGE LDS 4× sample buffer (Invitrogen) and 0.1 M DTT, samples were heated at 95°C for 10 min. The Odyssey Protein Weight Marker was used as a size reference (LI-COR, Lincoln, NE, USA). Proteins were separated by SDS-PAGE (NuPAGE 10% Bis-Tris precast gel and MES SDS running buffer (Invitrogen) and transferred to a nitrocellulose membrane (Protran, Schleicher & Schuell, Dassel, Germany) using NuPAGE transfer buffer. After blocking for 2 hours with PBS containing 5% Protifar (Nutricia, Schiphol, The Netherlands) and 0.5% bovine serum albumin, the blot was incubated with OP10L anti-c-Myc antibody to detect MYC-tagged PDE8A (1∶5000; Calbiochem, San Diego, CA, USA), and SC-1616 anti-β-actin antibody (1∶200; Santa Cruz Biotechnology, Santa Cruz, CA, USA) to detect β-actin. IRDye 800CW conjugated Goat anti-Mouse IgG (1∶15000; 926-32210, LI-COR, Lincoln, NE, USA) and IRDye 680LT conjugated Donkey anti-Goat IgG (1∶15000; 926–32224, LI-COR) were used as secondary antibodies to visualize expression using the Odyssey infrared image system (LI-COR). Image J software was used to quantify protein expression.

### miRNA target selection

Selected microRNAs that were predicted to target PDE8A mRNA were identified with the microRNA Target Filter tool from IPA (Ingenuity Systems Inc., Redwood City, CA, USA). Expression of miRNAs in monocytes and macrophages was characterized by next-generation sequencing with the SOLiD system and qPCR, EMBL-EBI ArrayExpress public database (http://www.ebi.ac.uk/arrayexpress/) accession number: EMTAB-1969 [43]. miRNA expression from 1 donor was paired with the mean expression of PDE8A from 2 donors obtained from gene expression data in similar samples (GSE35495), SubSeries GSE49240 (samples GSM1195728–39), part of the SuperSeries GSE35495 [47]; miRNAs were selected if expression levels were lower in macrophages, compared to monocytes.

### Statistical analysis

For statistical analysis the paired and unpaired T-test or the mixed linear model, repeated covariance: diagonal were used (IBM SPSS Statistics 20).

## Supporting Information

Figure S1 (A) Western Blot analysis of HEK293T cells in which the efficiency of the lentiviral vectors containing shRNAs against an MYC-tagged PDE8A or a control shRNA was determined. (B) Efficiency of *PDE8A* mRNA downregulation was analyzed by transduction of macrophages with lentiviral vectors expressing shRNAs against *PDE8A* or a control shRNA. *PDE8A* mRNA expression was determined 2 days post-transduction by qPCR. Statistical differences were analyzed using a paired T test.
(TIF)

Table S1 Primers used for qPCRs and cloning.
(DOCX)
