# Supplementary figures and images for: Phosphodiesterase 8a Supports HIV-1 Replication in Macrophages at the Level of Reverse Transcription
Source: PLoS One. 2014 Oct 8;9(10):e109673. doi: 10.1371/journal.pone.0109673 (PMC4190361; doi:10.1371/journal.pone.0109673)

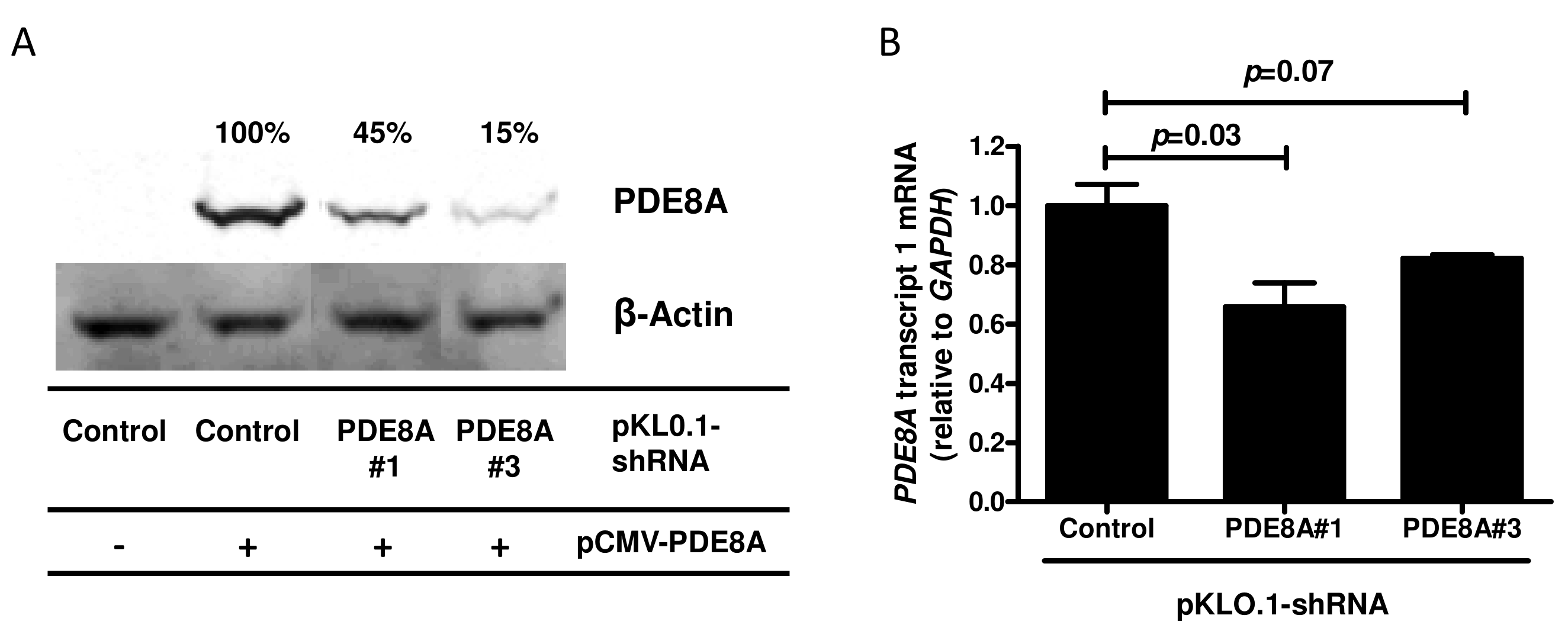

Supplement: Figure S1 — (A) Western Blot analysis of HEK293T cells in which the efficiency of the lentiviral vectors containing shRNAs against an MYC-tagged PDE8A or a control shRNA was determined. (B) Efficiency of PDE8A mRNA downregulation was analyzed by transduction of macrophages with lentiviral vectors expressing shRNAs against PDE8A or a control shRNA. PDE8A mRNA expression was determined 2 days post-transduction by qPCR. Statistical differences were analyzed using a paired T test. (TIF) [file pone.0109673.s001.tif]
