# Supplementary material for: Phosphodiesterase 8a Supports HIV-1 Replication in Macrophages at the Level of Reverse Transcription
Source: PLoS One. 2014 Oct 8;9(10):e109673. doi: 10.1371/journal.pone.0109673 (PMC4190361; doi:10.1371/journal.pone.0109673)
Supplement: Table S1 — Primers used for qPCRs and cloning. (DOCX) [file pone.0109673.s002.docx]

Supplementary Table 1: Primers used for qPCRs and cloning

| **Name** | **Sequence (5’- 3’)** | **Target** |
| --- | --- | --- |
| FW-GAPDH* | CGAGCCACATCGCTCAGACACC | *GAPDH* |
| REV-GAPDH* | CAAATGAGCCCCAGCCTTCTCCATG | *GAPDH* |
| FW-PDE8A* | CGTTTTATACAGTATGCAAATCCT | *PDE8A* |
| REV-PDE8A* | GCTTTGACGTCTAGTGAGCC | *PDE8A* |
| Fw-miR-145-5p* | GTCCAGTTTTCCCAGGAATCCCT | miR-145-5p |
| B-actin-S* | GGGTCAGAAGGATTCCTATG | *β-actin* |
| B-actin-AS* | GGTCTCAAACATGATCTGGG | *β-actin* |
| eRT2-F* | GTGCCCGTCTGTTGTGTGAC | R/U5 |
| eRT2-R* | GGCGCCACTGCTAGAGATTT | R/U5 |
| eRT2-P* | (FAM)-CTAGAGATCCCTCAGACCCTTTTAGTCAGTGTG G-(TAMRA) | R/U5 |
| Pol-B-02* | CTTCTAAATGTGTACAATCTAGTTGCC | Pol |
| Pol-E-03* | TGATTTTAACCTGCCACCTGTAGTAG | Pol |
| Pol-P* | (FAM)-CTGTGATAAATGTCAGCTAAAAGGAGAAGCCA-(TAMRA) | Pol |
| HIV-1-LTR-R-FW | ATGCCACGTAAGCGAAACTGCTGGCTAACTAGGGAACCCACTG | R |
| Alu-REV | TCCCAGCTACTGGGGAGGCTGAGG | *Alu* |
| Marker-FW | ATGCCACGTAAGCGAAACTG | U5 |
| HIV-1-LTR-U5-REV | CACACTGACTAAAAGGGTCTGAGG | U5 |
| FW-miR145-5p-BamHI^#^ | GATAGGATCCCACCCTGGCTGCTACAGAT | miR-145-5p |
| REV-miR145-5p-KpnI^#^ | CTTAGGTACCCTCCAGGGACAGCCTTCTTC | miR-145-5p |
| FW-miR145-target-KpnI^#^ | GACAGGTACCGACACTTTTACTGCACTATAG | *PDE8A-3’UTR* |
| REV-miR145-5p-target-KpnI^#^ | CTATGGTACCGAAGCAGCCTTCTTTAGCAC | *PDE8A-3’UTR* |

* Primers used for qPCR

^#^ Primers used for cloning

^$^ Marker sequence underlined
